# Synthesis of Kisspeptin-Mimicking Fragments and Investigation of their Skin Anti-Aging Effects

**DOI:** 10.3390/ijms21228439

**Published:** 2020-11-10

**Authors:** Kyung-Eun Lee, Sugyeong Jeong, Seok Kyun Yun, Seoyeon Kyung, Abadie Sophie, Sang Hyun Moh, Hyo Hyun Seo, Myeong Sam Park, Seunghyun Kang, Hyeonju Yeo

**Affiliations:** 1 COSMAX BTI R&I Center, Material Lab, Pangyo Inno Valley E 255, Pangyo-ro, Bundang-gu, Seongnam-si, Gyeonggi-do 13486, Korea; leeke@cosmax.com (K.-E.L.); sgjeong@cosmax.com (S.J.); sanzuk@cosmax.com (S.K.Y.); sykyung@cosmax.com (S.K.); msampark@cosmax.com (M.S.P.); shyunk@cosmax.com (S.K.); 2 Syntivia, Centre Pierre Potier, 1 place Pierre Potier, Entrée BP 50624, 31106 Toulouse, France; sabadie@syntivia.fr; 3 BIO-FD&C Co., Smart Valley A-510, 30 Songdomirae-ro, Yeonsu-gu 21990, Korea; biofdnc@gmail.com (S.H.M.); hhseo@biofdnc.com (H.H.S.)

**Keywords:** Kisspeptin-mimicking fragments, 11β-HSD1, collagen, skin aging, MMP-1

## Abstract

In recent years, a number of active materials have been developed to provide anti-aging benefits for skin and, among them, peptides have been considered the most promising candidate due to their remarkable and long-lasting anti-wrinkle activity. Recent studies have begun to elucidate the relationship between the secretion of emotion-related hormones and skin aging. Kisspeptin, a neuropeptide encoded by the *KISS1* gene, has gained attention in reproductive endocrinology since it stimulates the reproductive axis in the hypothalamus; however, the effects of Kisspeptin on skin have not been studied yet. In this study, we synthesized Kisspeptin-10 and Kisspeptin-E, which are biologically active fragments, to mimic the action of Kisspeptin. Next, we demonstrated the anti-aging effects of the Kisspeptin-mimicking fragments using UV-induced skin aging models, such as UV-induced human dermal fibroblasts (Hs68) and human skin explants. Kisspeptin-E suppressed UV-induced 11 beta-hydroxysteroid dehydrogenase type 1 (11β-HSD1) stimulation leading to a regulation of skin aging related genes, including type I procollagen, matrix metalloproteinases-1 (MMP-1), interleukin-6 (IL-6), and IL-8, and rescued the skin integrity. Taken together, these results suggest that Kisspeptin-E could be useful to improve UV-induced skin aging by modulating expression of stress related genes, such as 11β-HSD1.

## 1. Introduction

Skin aging is a complex biological process influenced by a combination of intrinsic and extrinsic factors [1]. Among various extrinsic factors, the psychological perception of pressure, which arises when people are under mental, physical, or emotional pressure, has been known to affect the biological mechanisms involved in the aging process, including in the skin [2]. In response to psychological stress, the hypothalamic–pituitary–adrenal (HPA) axis coordinates the production and secretion of glucocorticoids (GC) and these steroid hormones are further regulated by 11 beta-hydroxysteroid dehydrogenase type 1 (11β-HSD1), which catalyzes the conversion of inactive cortisone to active cortisol [3,4]. Cortisol is a kind of stress hormone that regulates a wide range of stress responses in several tissues by binding to the glucocorticoid receptor (GR) and translocating to the nucleus. It has been reported that the activated glucocorticoids decrease collagen content, which are degraded by matrix metalloproteinases (MMPs), and act as negative regulators in the process of dermal fibroblast proliferation [5]. In addition, the elevated expression and activity of 11β-HSD1 lead to an increase in GCs in aged and photo-exposed skin [6,7]. Furthermore, 11β-HSD1 deficient mice showed the improvement of collagen density as well as the expression of collagen synthesis-related genes. Emerging evidence indicates that the inhibition of 11β-HSD1 may facilitate the improvement of age-related impairments in dermal collagen content and wound healing [5,6].

The peptides have gained much attention as the functional ingredient candidates related to skin care, with the function of the cell migration, proliferation, inflammation, melanogenesis, as well as the synthesis and regulation of proteins [8]. On that note, it has been reported that various synthetic peptides have been applied as topical ingredients to induce specific biological activities such as skin anti-aging effect. For instance, the synthetic peptide of t type I procollagen fragment with the amino acid sequence of lysine-threonine-threonine-lysine-serine (KTTKS) showed to increase extracellular matrix production in fibroblasts [9]. Other study also has been reported that the substance P is a neuropeptide composed of 11 amino acids and the treatment of SP hydrogel enhanced the expression of type I procollagen whereas decreased the MMP-1 expression, thus showing its potential anti-aging effect in fibroblasts [10].

Kisspeptin is a neuropeptide encoded by the *KISS1* gene that binds with the G-protein coupled receptor 54 (also known as the KISS1 receptor) ligand. The several studies have revealed that Kisspeptin is an upstream key mediator of pulsatile and surge GnRH releases that play an integral role in female reproduction, including puberty onset, cerebral differentiation, ovulation, and regulating reproductive metabolism [11,12,13]. However, several studies for the safety of Kisspeptin treatment in human have been reported that the Kisspeptin treatment showed no changes in blood pressure or pulse rate as well as no adverse events or other side effects in human body [14,15]. The initial protein product of the *KISS1* gene is a 145-amino acid peptide with an arginine phenylalanine (RF) amide terminal group, and it can be cleaved into shorter and biologically active peptides known as Kisspeptin-54, Kisspeptin-14, Kisspeptin-13, and Kisspeptin-10 [16,17]. The well-known active fragment, Kisspeptin-10 (Kisspeptin 45-54), has been proven to show the activity of full length of kisspeptin in various types of experiments. The previous study has been reported that the Kisspeptin 45-50 (amino acids of YNWNSF) plays the key role and also successfully bound with amyloid-β, prion protein, and amylin, showing neuroprotective efficacy [18]. It was reported that Kisspeptin is expressed in diverse tissues, including the pancreas, adipose tissue, gonads, and placenta [19,20]; however, its main functional role is mediated by its expression within the central nervous system. In a recent study, Dhillo and his colleagues found the effects of Kisspeptin on limbic brain activity and behavior by demonstrating that Kisspeptin administration enhanced the limbic brain activity in response to sexual and couple-bonding stimuli. The enhancement of Kisspeptin in limbic brain structures correlates with psychometric measures of reward, drive, mood, and sexual aversion, providing functional significance [21]. Although some existing literature reported that neuropeptides are believed to be related to skin aging, the mechanism of Kisspeptin for skin cell aging has not been thoroughly discussed, yet [1,2].

In this study, to mimic the action of Kisspeptin, we synthesized Kisspeptin-10 (Kisspeptin 45-54, the amino acid sequence: Tyr–Asn–Trp–Asn–Ser–Phe–Gly–Leu–Arg–Phe–NH_2_), which is known as biological active fragments of Kisspeptin. Kisspeptin-E (Kisspeptin 45-49, the amino acid sequence: Tyr–Asn–Trp–Asn–Ser–NH_2_), was synthesized using solid-phase peptide synthesis that have potential to be biologically active fragments similar to the Kisspeptin-10. They were further precipitated and isolated using high performance liquid chromatography (HPLC). To investigate the role of Kisspeptin-mimicking peptides on skin aging, here we examined the mRNA expression levels of the 11β-HSD1 enzyme and skin aging-related genes, such as MMP-1. Furthermore, we evaluated the effects of Kisspeptin-mimicking peptides on dermal protein expressions including collagen, MMP-1, and 11β-HSD1 and epidermis thickness in human skin explants. Our findings point toward the Kisspeptin-mimicking peptides being important for the improvement skin integrity, thus providing insight into its functional contribution to skin anti-aging.

## 2. Results

### 2.1. Characterization of Kisspeptin-Mimicking Peptides

**Scheme 1:**
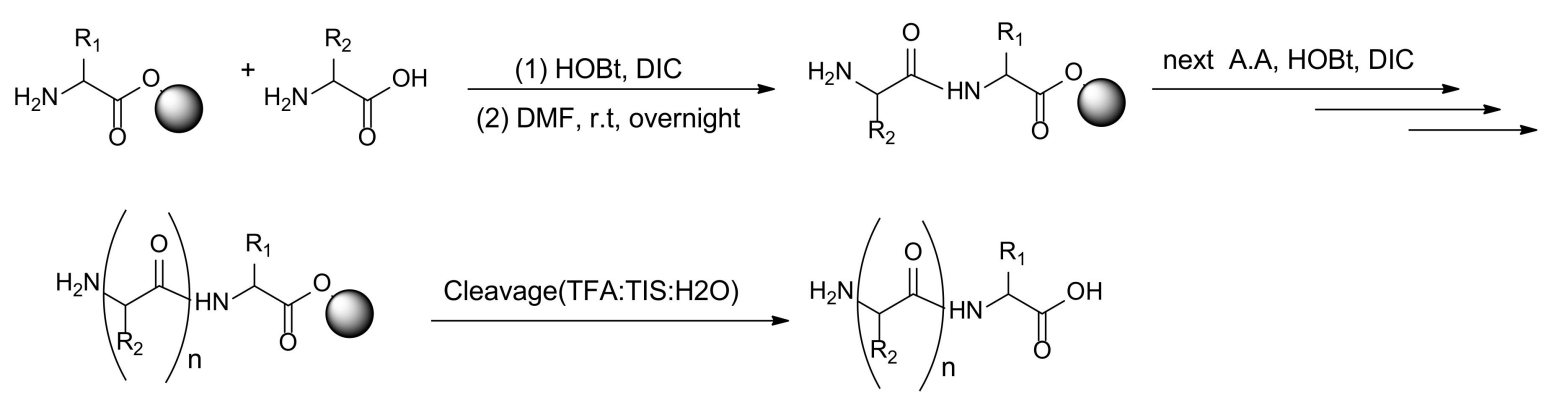
Scheme of solid-phase peptide synthesis of Kisspeptin-mimicking bioactive products.

**Figure 1:**
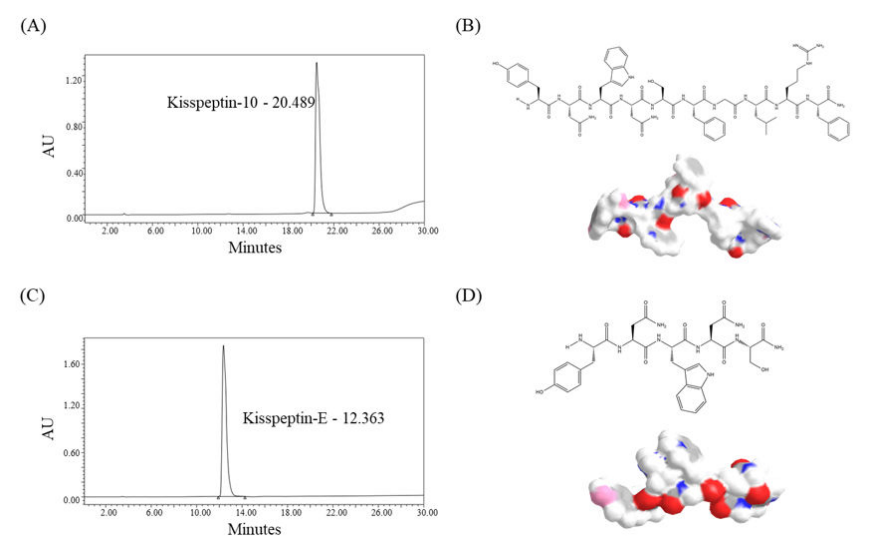
Characterization of Kisspeptin-mimicking peptides. The HPLC elution times were measured to confirm (**A**) Kisspeptin-10 and (**C**) Kisspeptin-E. The chemical structures and 3D rendering of the mimicking peptides represent (**B**) Kisspeptin-10 and (**D**) Kisspeptin-E.

The Kisspeptin-mimicking peptides were synthesized using the solid-phase peptide synthesis method (Scheme 1) and successfully cleaved from the resin. Then, the peptides were characterized by HPLC chromatogram (Figure 1). The actual molecular weights (MWs) of newly synthesized Kisspeptin-10 (theoretical MW: 1302.44 g/mol) and Kisspeptin-E (theoretical MW: 681.70 g/mol) were characterized by matrix assisted laser desorption/ionization time-of-flight mass spectrometry (MALDI-TOF) mass, defined as 1301.63 g/mol and 681.29 g/mol, respectively, indicating that the synthesis of Kisspeptin-mimicking fragments was successful (Figure 1A,B). The chemical structure and 3D rendering of Kisspeptin-10 with the amino acid sequence of Tyr–Asn–Trp–Asn–Ser–Phe–Gly–Leu–Arg–Phe–NH_2_ was represented by the chemical formula C_63_H_83_N_17_O_14_ and Kisspeptin-E with the amino acid sequence of Tyr–Asn–Trp–Asn–Ser–NH_2_ was represented by the chemical formula C_31_H_39_N_9_O_9_ (Figure 1C,D).

### 2.2. Inhibitory Effects of Kisspeptin-Mimicking Peptides on 11β-HSD1 Expression

**Figure 2:**
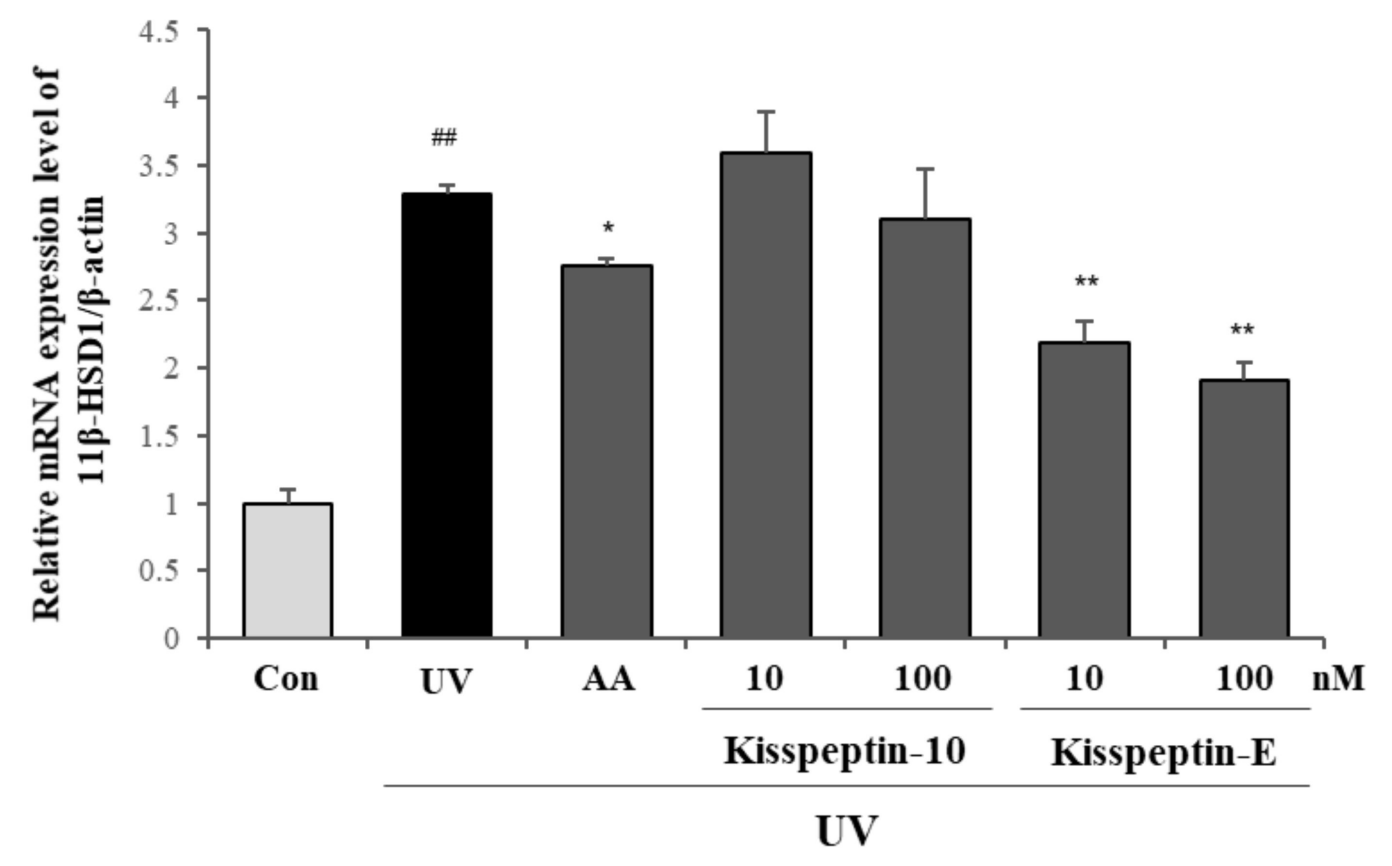
Kisspeptin-mimicking peptides inhibited the mRNA expression of 11β-HSD1 in UVB-induced Hs68 fibroblasts. Kisspeptin-10 and Kisspeptin-E were treated in UVB-irradiated Hs68 fibroblasts. The mRNA expression level of 11β-HSD1 was measured by RT-qPCR. The means ± SEs are the average of three independent experiments. AA, Ascorbic acid; ^##^ *p* < 0.01 indicates a significant difference from the control. * *p* < 0.05 and ** *p* < 0.01 indicate a significant difference from the UVB-induced control.

We and other groups previously reported that the expression levels of 11β-HSD1 are elevated in photo-exposed skin tissue and that its mRNA expression levels are also increased in photo-exposed dermal fibroblasts [6,7]. Here, we aimed at analyzing the effects of Kisspeptin-mimicking peptides on the mRNA expression levels of 11β-HSD1, an enzyme that converts inactive cortisone into active cortisol, in Hs68 fibroblasts. As shown in Figure 2, the mRNA expression levels of 11β-HSD1 were significantly decreased in Kisspeptin-E treated Hs68 fibroblasts whereas no changes in Kisspeptin-10 treated cells were observed. Given that Kisspeptin-E functions as a major inhibitor of 11β-HSD1, it is conceivable that Kisspeptin-E may contribute to a stronger interaction with 11β-HSD1 than Kisspeptin-10 due to its lesser amino acid molecular structure. It is of interest that the treatment of Kisspeptin-E dramatically decreased the expression of 11β-HSD1, even compared to ascorbic acid (AA) treatment, suggesting that Kisspeptin-E may engage the gene expression of 11β-HSD1 to lead to anti-photo aging effects.

### 2.3. The Effects of Kisspeptin-Mimicking Peptides on MMP-1 and Type I Procollagen Expression

**Figure 3:**
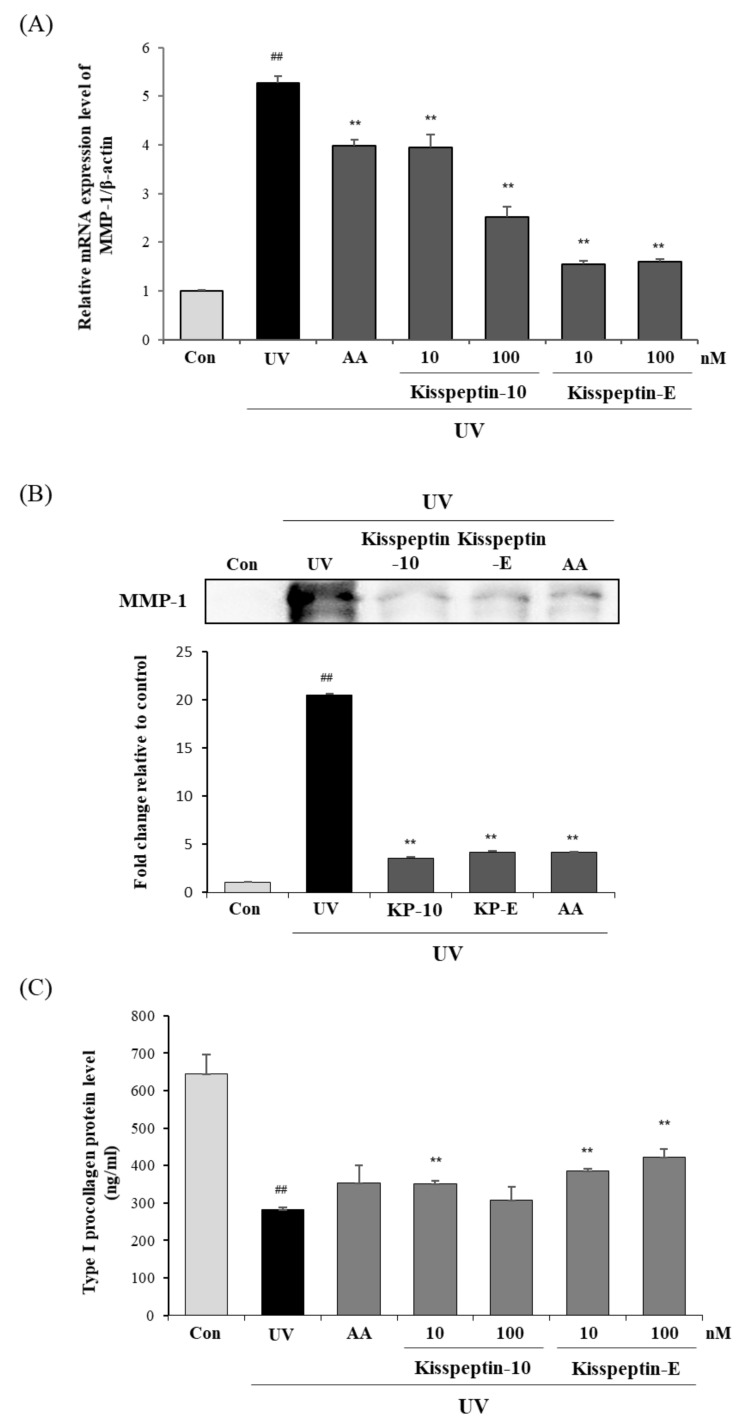
Kisspeptin-mimicking peptides decreased the mRNA and protein expression of MMP-1 whereas increased the production of type I procollagen in UVB irradiated Hs68 fibroblasts. The Hs68 fibroblasts were treated with Kisspeptin-10 and Kisspeptin-E for 24 h after UVB irradiation. AA was used as a positive control (10 μg/mL). The mRNA (**A**) and protein (**B**) expression of MMP-1 were measured by RT-qPCR and immunoblot, respectively. (**C**) The type I procollagen protein secretion was measured by ELISA assay. The means ± SEs are the average of three independent experiments. KP-10, Kisspeptin-10; KP-E, Kisspeptin-E; AA, Ascorbic acid; ^##^ *p* < 0.01 indicates a significant difference from the control. ** *p* < 0.01 indicates a significant difference from the UVB-induced control.

It was previously reported that Kisspeptin negatively regulated the mRNA expression levels and protein activity of MMP-9 in HT-1080 cells [22]. In order to determine whether Kisspeptin-mimicking peptides regulate MMP-1, the main collagenase, expression, we utilized well-established UVB irradiated fibroblasts to induce skin photo-aging. The Hs68 fibroblasts were irradiated with UVB and treated with Kisspeptin-mimicking peptides or AA for 24 h. The treatment of Kisspeptin-10 or Kisspeptin-E significantly inhibited the UVB-induced mRNA and protein expression levels of MMP-1, compared to UVB untreated cells (Figure 3A,B). Notably, Kisspeptin-E inhibited the MMP-1 mRNA expression levels more than Kisspeptin-10. In addition, Kisspeptin-E treatment increased type I procollagen protein secretion in a dose-dependent manner (Figure 3C). Our results provide evidence that Kisspeptin-mimicking peptides, especially Kisspeptin-E, contribute to the protective effects against UVB-induced skin collagen degradation by suppressing MMP-1 gene expression.

### 2.4. Inhibitory Effects of Kisspeptin-Mimicking Peptides on IL-6 and IL-8 Expression

**Figure 4:**
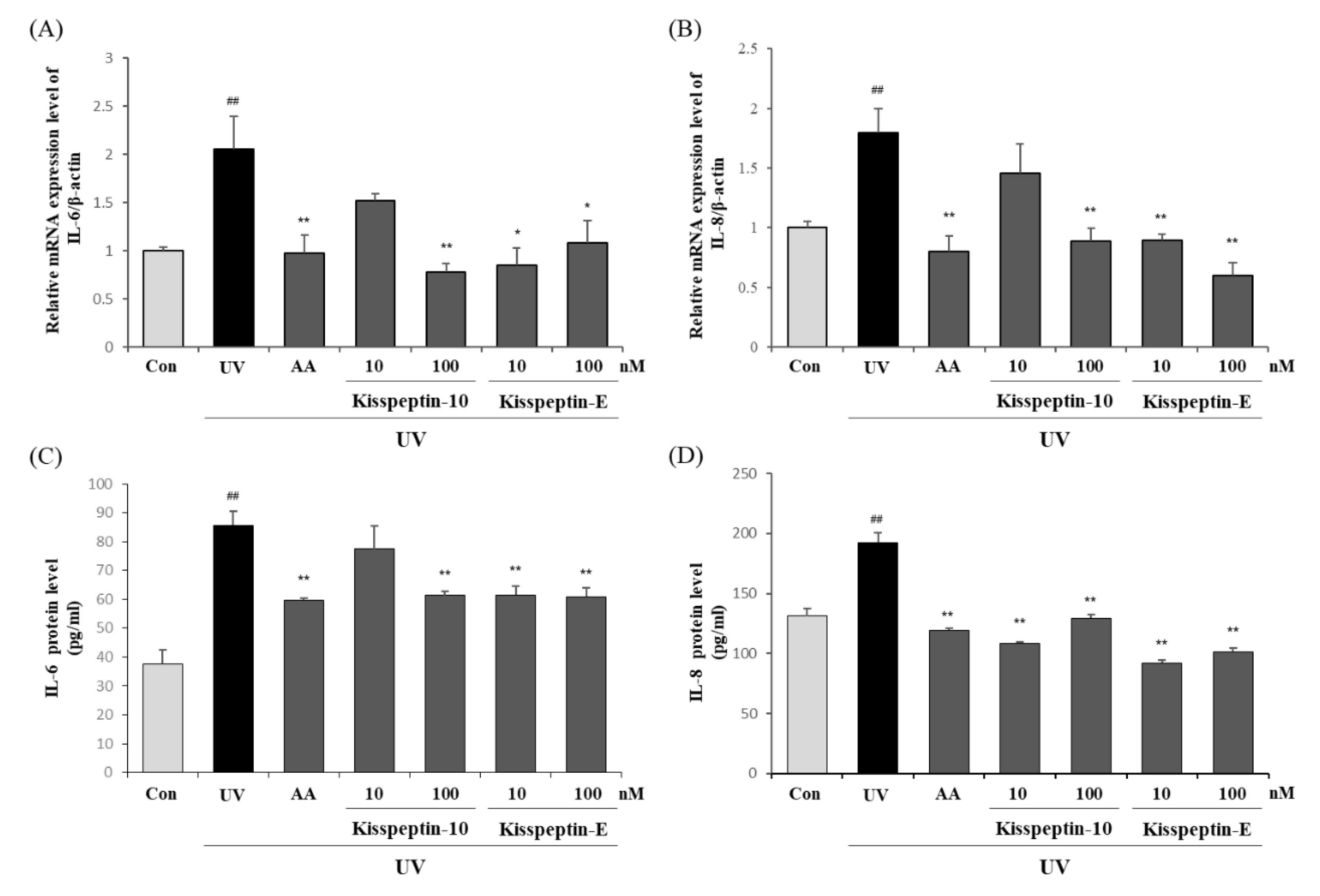
Kisspeptin-mimicking peptides decreased mRNA and protein expressions of IL-6 and IL-8 in UVB irradiated Hs68 fibroblasts. The Hs68 fibroblasts were treated with Kisspeptin-10 and Kisspeptin-E for 24 h after UVB irradiation. The mRNA expression (**A**,**B**) and protein secretion (**C**,**D**) of IL-6 and IL-8 were measured by RT-qPCR and ELISA. The means ± SEs are the average of three independent experiments. AA, Ascorbic acid; ^##^ *p* < 0.01 indicates a significant difference from the control. * *p* < 0.05 and ** *p* < 0.01 indicate a significant difference from the UVB-induced control.

Extensive studies have reported that UV irradiation contributes to skin aging and inflammation by increasing pro-inflammatory cytokines. Procollagens are expressed and combined to represent the dermal extracellular matrix (ECM) and are also known to be regulated by interleukin-6 (IL-6) and MMP-1 [23]. To verify the effects of Kisspeptin-mimicking peptides on pro-inflammatory cytokines, such as IL-6 and IL-8, Kisspeptin-10 and Kisspeptin-E were treated in UVB irradiated Hs68 fibroblasts and the mRNA and protein levels were evaluated. As shown in Figure 4, Kisspeptin-10 and Kisspeptin-E significantly decreased the mRNA expression and protein secretion of pro-inflammatory cytokines. These results suggest that Kisspeptin-mimicking peptides have anti-inflammatory properties.

### 2.5. Regulatory Effects of Kisspeptin-E on Dermis Proteins in Human Skin

Recently, it has been reported that UVA irradiation induced the epidermal disruption and alteration of dermal ECM integrity in a human skin explant model [24]. To support our observation, we confirmed to evaluate the skin anti-aging effects of Kisspeptin-E utilizing human skin explant models. Kisspeptin-E was selectively treated into the human skin explants with UVA irradiation for three days and harvested to evaluate the skin anti-aging related targets. The type I collagen, 11β-HSD1, and MMP-1 expression were proportional to the fluorescence intensity of the staining.

**Figure 5:**
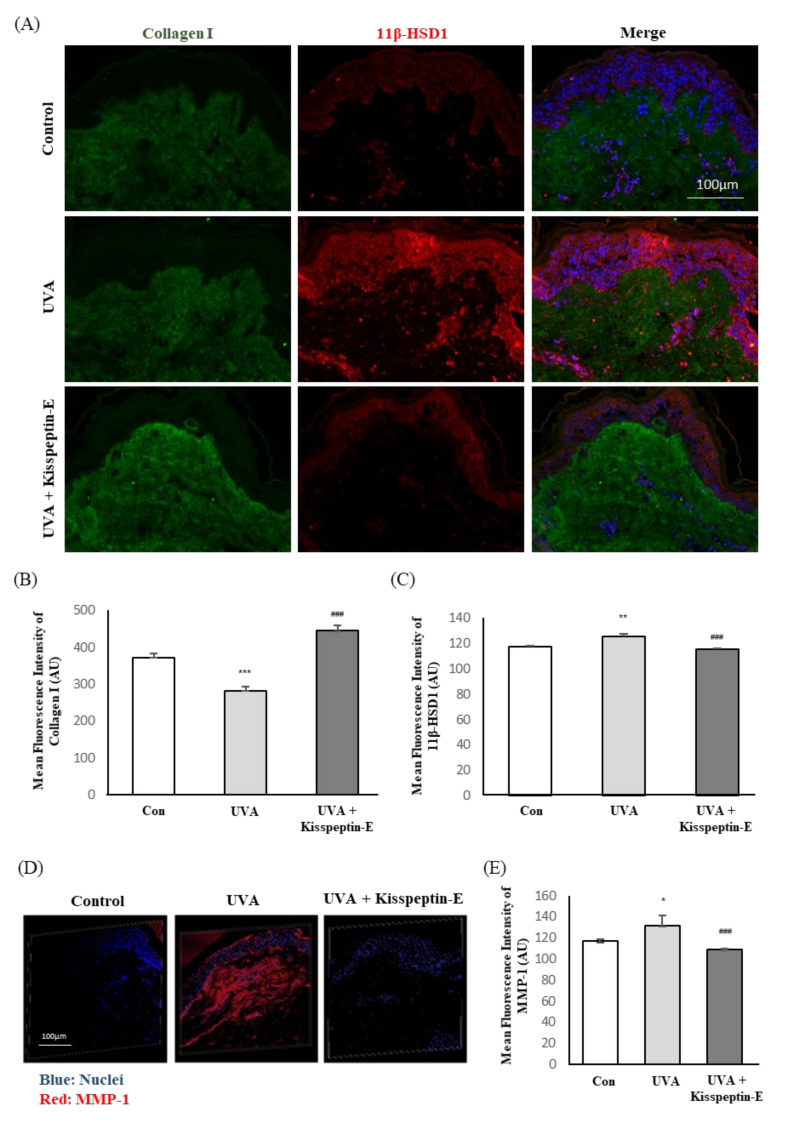
Kisspeptin-E regulated the 11β-HSD1, type I collagen, and MMP-1 protein expressions in the UVA irradiated human skin explant model. Human skin explants were topically treated with Kisspeptin-E before, during, and after UVA irradiation. The proteins of (**A**,**B**) type collagen I and (**A**,**C**) 11β-HSD1 were labeled by immunofluorescence and observed by fluorescence microscopy (DM5000B–Leica-Microsystems). Ten areas were observed by samples. (**C**,**E**) The measured fluorescence was quantified by image analysis (Image J). (**D**) The MMP-1 enzyme was labeled by immunofluorescence and observed by confocal microscopy (Zeiss LSM510 NLO). (**D**,**E**) The 3D representation and the mean fluorescence intensity of staining are shown. ^***^ *p* < 0.001, ** *p* < 0.01 indicates a significant difference from the control. ^###^ *p* < 0.001 indicates a significant difference from the UVA-irradiated control.

Consistent with the above observation, the amount of type I collagen was decreased up to −25% after UVA irradiation and Kisspeptin-E treatment rescued 43% more of it than the UVA irradiated skin explant. The UVA irradiation slightly increased the amount of 11β-HSD1 by +6.8% and Kisspeptin-E treatment significantly decreased by 8.2% compared to the UVA irradiated skin (Figure 5A–C). Additionally, UVA irradiation increased MMP-1 expression whereas the treatment of Kisspeptin-E decreased expression as shown by immunostaining in the 3D representation (Figure 5D).

### 2.6. Recovery Effects of Kisspeptin-E on Epidermis Thickness in Human Skin

**Figure 6:**
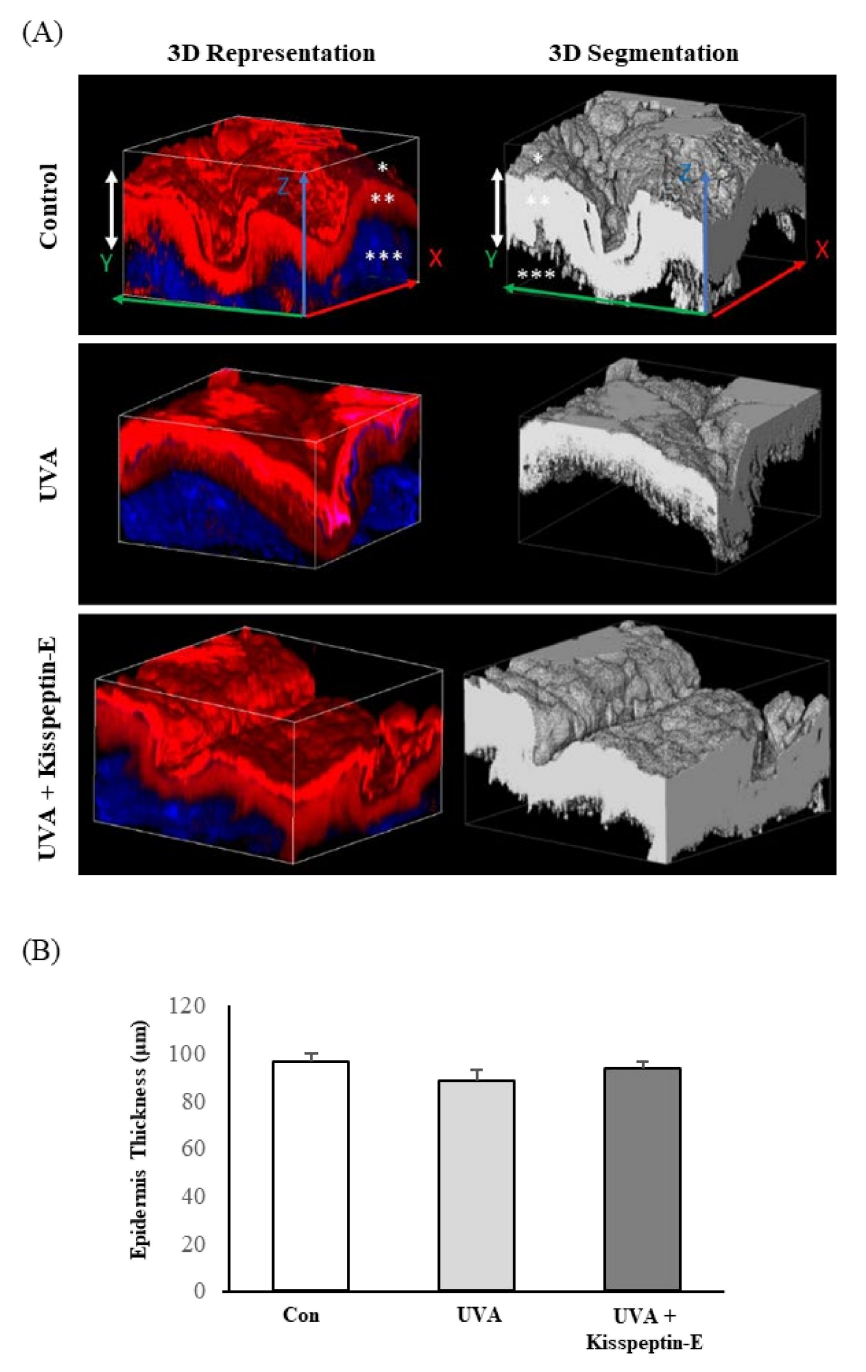
Kisspeptin-E recovered the epidermal thickness in UVA irradiated human skin explants. Human skin explants were topically treated with Kisspeptin-E and with UVA irradiation. (**A**) After treatment, human skin explants were fixed with formalin, and the cytoplasm of keratinocytes was labeled using a fluorescent cell tracker. Then, 10 z-stacks were acquired on each sample by confocal microscopy (epidermis in red, dermis in blue, white arrow is epidermis thickness, * stratum corneum, ** epidermis, ^***^ dermis) (Zeiss LSM510 NLO). We delimited and quantified the volume to extract the average thickness of the epidermis with Amira^®^ software (Thermo Fisher Scientific, Waltham, MA, USA). (**B**) The average thickness of 10 z-stacks ± SE at mean per conditions was calculated.

To evaluate the structural change as well as epidermis thickness, a 3-dimensional observation was performed by confocal microscopy directly on human skin explants after Kisspeptin-E treatment. The epidermis thickness of the control sample was around 92.6 µm (+/− 3.1µm). After UVA irradiation, the epidermis thickness decreased to 88.1 µm, up to −4.9%, but it was not significant. After UVA irradiation and Kisspeptin-E treatment, the epidermis thickness was recovered to 93.3 µm, similar to the control skin (Figure 6A). Therefore, it is reasonable to speculate that Kisspeptin-E would have protective and recovery effects against UVA irradiation.

## 3. Discussion

*KISS1* gene encodes the production of the 145 amino acid pro-peptide which is then processed to produce Kisspeptin-54 (known as Kisspeptin or metastin), and another three fragments sharing the same 10 amino acid sequences at the C-terminal (Kisspeptin-14, Kisspeptin-13 and Kisspeptin 10). This gene was originally discovered in Hershey (Pennsylvania) and named *KISS1* since the city was famous for producing chocolate ‘Kisses’ [11]. Kisspeptin is a hypothalamic neuropeptide and mainly plays governing the hypothalamic–pituitary–gonadal (HPG) axis by regulating the secretion of gonadotropin-releasing hormone (GnRH) [25]. Previous studies have shown that Kisspeptin and GPR54 signaling was key process for secretion of GnRH and gonadotrophin, thereby modulating reproductive activity such as puberty onset, cerebral differentiation, ovulation, and regulating reproductive metabolism [11,12,13,26,27]. Likewise, despite the diverse roles of Kisspeptin as a neuropeptide that are described above, less is known about the effect of Kisspeptin on skin and aging.

Recently, the emotion-related neuropeptides have been considered as one of the promising candidates to reduce stress and their relationship with skin aging has been extensively studied [1,2]. For example, oxytocin, the neuropeptide hormone associated with the emotion of happiness and complex social behavior, is known to reduce the stress levels by down-regulating the production of glucocorticoids [28]. It has been also reported that this neuropeptide controls cell differentiation and acts as a mediator in cutaneous homoeostasis, influencing proliferation of dermal fibroblasts and keratinocytes [29]. These studies have shown that Kisspeptin has functions in modulating sexual and emotional brain processing in humans. Thus, we hypothesized that Kisspeptin would affect psychological stress-related skin aging processes and evaluated the anti-skin-aging effect.

In this study, in order to elucidate the relationship between the neuropeptide and skin aging, we firstly synthesized the Kisspeptin-mimicking fragments and evaluated their effects on human dermal fibroblasts. Kisspeptin-10 (Tyr–Asn–Trp–Asn–Ser–Phe–Gly–Leu–Arg–Phe–NH_2_) and Kisspeptin-E (Tyr–Asn–Trp–Asn–Ser–NH_2_) were synthesized using the solid-state phase method as Kisspeptin-mimicking fragments. Then, we determined whether Kisspeptin-mimicking peptides functioned to decrease the 11β-HSD1 expression in Hs68 fibroblasts. 11β-HSD1 was identified as an enzyme interconverting cortisol and cortisone. 11β-HSD1 has been well known as a potent indicator of skin photo-aging since the activity and expression of 11β-HSD1 were increased in photo-exposed skin tissues. Prior studies have reported that stress inducers such as UV or IR increased 11β-HSD1 expression in human skin leading to a decrease in collagen content and 11β-HSD1 inhibitors prevented skin aging by regulating cortisol activation [7,30,31]. Moreover, 11β-HSD1 regulates cortisol levels and excessive cortisol production contributes to a reduction of dermal collagen content, as well as thins and flattens the dermal-epidermal junction, a hallmark of aging skin [6,32,33]. It has been demonstrated that the histological skin profile of aged 11β-HSD1 deficient mice appeared to be more similar in the young wild type littermates than the aged wild type littermates with improved collagen density and more orderly organization [6].

Our results demonstrated that Kisspeptin-E decreased UVB-induced mRNA expression levels of 11β-HSD1 in Hs68 fibroblasts (Figure 2). Subsequently, the treatment of Kisspeptin-mimicking peptides, especially Kisspeptin-E, inhibited the mRNA and protein expression of MMP-1, whereas increased type I procollagen secretion in UVB-induced Hs68 fibroblasts. In addition, Kisspeptin-mimicking peptides decreased the UVB-induced increase of IL-6 and IL-8 expression (Figure 3 and Figure 4). These data indicated that Kisspeptin-E, among the two mimicking peptides, prevented photo-induced dermal skin aging by mainly inhibiting 11β-HSD1 expression in Hs68 fibroblasts. Furthermore, the anti-aging effects of Kisspeptin-E on the human skin layer were evaluated using a human skin explant model. In agreement with our prior in vitro studies, the treatment of Kisspeptin-E showed a decrease in UVA-induced 11β-HSD1 and MMP-1 expression in the human skin explant model. Moreover, Kisspeptin-E rescued the collagen density that was reduced in the dermis by UV irradiation and improved epidermal thickness as well. These results demonstrated that Kisspeptin-E had function to inhibit the elevation of 11β-HSD1 by external stresses such as UV, thereby leading an improvement of skin integrity.

In conclusion, our results establish Kisspeptin-E as a new determinant of skin anti-aging effects. Kisspeptin-E, as a Kisspeptin-mimicking peptide, suppressed UV-induced 11β-HSD1 stimulation leading to a regulation of skin aging related genes and rescued the skin structure against UV irradiation. As resulted, we suggested that Kisspeptin-E may function to reduce the external stress-related skin aging. However, further studies are needed to determine whether Kisspeptin-E has a positive effect on skin under the direct psychological stress environments. Taken together, we conclude that both Kisspeptin-10 and Kisspeptin-E have skin anti-aging effects and, in particular, Kisspeptin-E has the ability to inhibit the gene expression of stress related skin aging.

## 4. Materials and Methods

### 4.1. Synthesis of Kisspeptin-Mimicking Peptides

Kisspeptin-10 was designed with the amino acid sequence of the Kisspeptin protein N-terminal from 112 to 121, assigned to be Tyr–Asn–Trp–Asn–Ser–Phe–Gly–Leu–Arg–Phe–NH_2_ and synthesized by the solid-phase peptide synthesis method (Scheme 1) [34]. The synthesis was conducted using Rink amide resin and amino acids with the protection of 9-fluorenylmethoxycarbonyl (FMOC). The amino acid residue was elongated after activation by N-hydroxybenzotriazole (HOBt) and N, N’-diisopropyl carbodiimide (DIC). For each step of amino acid elongation, amino acids, HOBt, and DIC in a 5-fold molar ratio to the resin were used. The reaction was carried out for 2 h at room temperature following by washing the resin with dimethylformamide (DMF) and dichloromethane (DCM) for 6 times, respectively. The product dried in ambient conditions and was then reacted with a solution containing trifluoroacetic acid (TFA):triisopropylsilane (TIS):H_2_O (90:5:5 (*v*/*v*)) to separate it from the resin. The product was further precipitated with cold ether, centrifuged, and purified by reverse-phase HPLC (column: Kromasil, C18, 5 μm, 110 Å, 250 × 21.2 mm). The aforementioned procedures were performed to produce Kisspeptin-E with the assigned amino acid sequence of Tyr–Asn–Trp–Asn–Ser–NH_2_.

### 4.2. Characterization of Kisspeptin-Mimicking Peptides

HPLC (Waters 650E advanced protein purification system equipped with Waters 616 pump and Waters 996 photodiod array detector) and matrix assisted laser desorption–time of flight (MALDI-TOF; Voyager-DE STR BioSpectrometry Workstation) instruments were used to characterize the as-prepared Kisspeptin-mimicking peptides.

### 4.3. Cell Culture and UVB Irradiation

The human dermal fibroblast (Hs68) cell line was purchased from the American Type Culture Collection (ATCC; Menassas, VA, USA). The cells were cultured in Dulbecco’s modified Eagle’s medium supplemented with 1% Antibiotic Antimycotic Solution (DMEM; HyClone Laboratories, Inc., Logan, UT, USA.) and 10% fetal bovine serum in an atmosphere of 5% CO_2_ at 37 °C. The cells were seeded in 6-well plates and washed with 1.5 mL Dulbecco’s phosphate buffered saline (DPBS), and the culture medium was replaced after 24 h incubation. After the Hs68 fibroblasts reached 80% confluence, the cells were rinsed with DPBS and then exposed to a 12 mJ/cm^2^ dose of UVB irradiation supplied by a CL-1000M UV Crosslinker (UVP, Upland, CA, USA) at a wavelength of 302 nm to induce photoaging responses. After UVB exposure, the cells were treated with serum-free DMEM or DMEM containing Kisspeptin-10 and Kisspeptin-E (10 or 100 nM).

### 4.4. RT-qPCR

Total RNA was isolated from cell pellets using Trizol reagent and quantified by spectrophotometry. The cDNA was synthesized in a 20 µL reaction containing 2 µg of total RNA, oligo (dT), and Reverse Transcription Premix under the following reaction conditions: 45 °C for 45 min and 95 °C for 5 min. Gene expression signals were quantified using real-time RT-PCR. The data were analyzed using the StepOne Plus^TM^ system software (Applied Biosystems, Foster City, CA, USA). RT-qPCR amplifications were performed using SYBR Green PCR Master Mix with premixed ROX (Applied Biosystems, Foster City, CA, USA) and primers (Bioneer, Daejeon, Korea) in an ABI 7300 following the manufacturer’s protocol. Reaction conditions were as follows: initiation at 95 °C for 10 min, followed by cycling conditions of 95 °C for 15 sec, 55 °C for 30 sec, and 72 °C for 30 sec for 40 cycles. The expression of β-actin was used as an internal control.

### 4.5. Enzyme-Linked Immunosorbent Assay (ELISA)

The Hs68 cell line was cultured in a 6-well plate for 24 h. The cells were then washed with phosphate buffered saline (PBS) and irradiated with UVB (15 mJ/cm^2^) through a thin layer of PBS. After UVB exposure, cells were incubated with serum-free DMEM containing Kisspeptin-10 and Kisspeptin-E (10 and 100 nM) for 24 h. Cell culture medium was collected after 24 h, and the production of IL-6, IL-8 (Invitrogen, Waltham, MA, USA), and type I procollagen (Takara Bio Inc., Otsu, Japan) were quantified using enzyme immunoassay kits.

### 4.6. Immunoblotting Analysis

The cell harvest was performed using radioimmunoprecipitation assay (RIPA) lysis buffer (Sigma–Aldrich; St Louis, MO, USA), and concentrations of the protein were determined using the Bradford assay. The levels of secreted protein were determined using culture media extract. Approximately 40 µg of each sample was separated by SDS-PAGE (10% acrylamide) and then transferred into a nitrocellulose membrane (Bio-Rad; Hercules, CA, USA). The membrane was blocked with 5% skim milk for 30 min and incubated overnight at 4 °C with primary antibodies against MMP-1 and β-actin (Abcam, Cambridge, MA, USA) overnight. After washing with tris-buffered saline with tween 20 (TBST) 3 times, the membrane was probed with a horseradish peroxidase-conjugated secondary antibody (Bethyl Laboratories, Montgomery, TX, USA) for 1 h at 4 °C. The signals were measured using an ECL Western blotting detection kit (Thermo Scientific; Waltham, MA, USA) and visualized with a G: Box Chemi System (Syngene; Cambridge, UK). The quantification was measured using Syngene Tools Gel image analysis.

### 4.7. Human Skin Explants and UVA Irradiation

Human skin tissue (NativeSkin^®^, Genoskin, Toulouse, France) was obtained from abdominoplasty of 1 skin donor (in triplicata), a woman, aged 38 years, with a phototype 2 on the Fitzpatrick scale. After the surgery, the skin was conserved for 24 h at 4 °C and transformed into a living explant via punch biopsy for incorporation into a solid support and a nutrient matrix (NativeSkin^®^). The human skin explants were maintained in culture at 37 °C, 5% CO_2_, in an incubator for 5 days with specific medium, provided by the supplier, renewed daily. The skin explants were treated 24 h before irradiation with Kisspeptin-E at 1 µM. Then, the skin explants were treated and irradiated with 60 J/cm^2^ of UVA once a day for three days (Biolink^®^ UVA lamp, Vilbert Lourmat, Marne la vallée, France, peak at 365 nm). Samples were also treated 24 h after the last irradiation and were collected. The negative control sample was human skin explants that were not irradiated (maintained in the irradiator but protected with aluminum).

### 4.8. Immunostaining in Human Skin

Paraffined skin sections were deparaffinized and hydrated. The antigen retrieval was performed in citrate at pH 6 at 90°C for 40 min. Sections were blocked with PBS containing 2% bovine serum albumin for 30 min and incubated with the primary antibodies anti-collagen 1 (Abcam, Massachusetts, VA, USA), anti-11β-HSD1 (Novus Biological, Centennial, CO, USA), or MMP-1 (Thermofisher, Waltham, MA, USA) overnight at 4 °C. A secondary antibody, Alexa Fluor 594 anti-rabbit or anti-mouse (Invitrogen Life Technologies, Carlsbad, CA, USA), was then added for detection. Nuclei were stained with 4,6-diamidino-2-phenylidole (DAPI, Sigma–Aldrich, St Louis, MO, USA) and slides were observed with a fluorescence microscope (DM5000B, Leica, Wetzlar, Germany). The mean fluorescence intensity was determined by image analysis (Image J) from 10 images for each sample.

### 4.9. Measurements of Epidermis Thickness

After treatment, skin explants were fixed with formalin, and the cytoplasm of keratinocytes was labeled using a fluorescent cell tracker (CMTMR™ orange, Thermofisher, Waltham, USA). Samples were cleared to make the skin transparent with Murray’s method. The transparent samples were placed directly under the microscope and acquisitions were performed. Then, 10 z-stacks were acquired from each sample by Confocal microscope (Zeiss LSM510 NLO, Carl Zeiss, Oberkochen, Germany). We delimited and quantified the volume to extract the average thickness of epidermis with Amira^®^ software (Thermo Fisher Scientific, Waltham, MA, USA). The average thickness of 10 z-stacks ± SE of the mean per condition was calculated.

## Abbreviations

AA	Ascorbic acid
ATCC	American Type Culture Collection
DCM	Dichloromethane
DIC	N, N’-diisopropyl carbodiimide
DMEM	Dulbecco’s modified Eagle’s medium
DMF	Dimethylformamide
DMSO	Dimethylsulfoxide
DPBS	Dulbecco’s phosphate buffered saline
ECM	Extracellular matrix
ELISA	Enzyme-linked immunosorbent assay
FMOC	9-fluorenylmethoxycarbonyl
GC	Glucocorticoids
GnRH	Gonadotropin-releasing hormone
GR	Glucocorticoid receptor
HOBt	N-hydroxybenzotriazole
HPA	Hypothalamic-pituitary-adrenal
HPG	Hypothalamic-pituitary-gonadal
HPLC	High performance liquid chromatograph
IL-6	Interleukin-6
MALDI-TOF	Matrix Assisted Laser Desorption/Ionization Time-of-Flight Mass Spectrometry
MMP-1	Matrix metalloproteinase-1
MW	Molecular weight
PBS	Phosphate buffered saline
RIPA	Radioimmunoprecipitation assay
RF	Arginine phenylalanine
RT-qPCR	Quantitative real-time polymerase chain reaction
TBST	Tris-Buffered Saline with Tween 20
UV	Ultraviolet
11β-HSD1	11 beta-hydroxysteroid dehydrogenase type 1
